# Morning free and total testosterone in HIV-infected men: implications for the assessment of hypogonadism

**DOI:** 10.1186/1742-6405-11-6

**Published:** 2014-01-22

**Authors:** Anne K Monroe, Adrian S Dobs, Frank J Palella, Lawrence A Kingsley, Mallory D Witt, Todd T Brown

**Affiliations:** 1 Division of General Internal Medicine, Johns Hopkins University School of Medicine, 1830 E Monument Street, Room 8060, Baltimore, MD 21287, USA; 2 Division of Endocrinology and Metabolism, Johns Hopkins University School of Medicine, Baltimore, MD USA; 3 Division of Infectious Diseases, Feinberg School of Medicine of Northwestern University, Chicago, IL USA; 4 Department of Infectious Diseases and Microbiology, University of Pittsburgh Graduate School of Public Health, Pittsburgh, PA USA; 5 Los Angeles Biomedical Research Institute at Harbor UCLA Medical Center, Torrance, CA USA

**Keywords:** Testosterone, Sex hormone binding globulin, HIV, Hypogonadism

## Abstract

**Background:**

Hypogonadism is common among HIV-infected men, even among men receiving antiretroviral therapy (ART). Our objective in this study was to determine the prevalence of biochemical hypogonadism among HIV-infected men compared with HIV-uninfected controls. We also examined the use of free testosterone (FT) and total testosterone (TT) measurements in the assessment of biochemical hypogonadism in HIV-infected and –uninfected men.

**Methods:**

This was a cross-sectional analysis from the Multicenter AIDS Cohort Study (MACS). TT levels were measured from archived serum using liquid chromatography-tandem mass spectrometry. FT was calculated from TT and sex hormone-binding globulin (SHBG) (measured by radioimmunoassay) using the Vermeulen equation. Biochemical hypogonadism was defined as having low TT, low FT, or both.

**Results:**

Of 945 men in the MACS Cardiovascular Substudy, T assays were not performed in 89 because of insufficient/no stored serum (n = 18) or use of T replacement therapy (TRT) (n = 71). 530 men had morning (AM) T measurements; 364 (68.7%) were HIV-infected. The prevalence of biochemical hypogonadism was similar in HIV-infected (34/364 = 9.3%) and HIV-uninfected (12/166 = 7.2%) men. Prevalence of hypogonadism, when men on TRT (n = 71) were included in the group of hypogonadal men, was higher in HIV-infected (104/434 = 24.0%) compared with HIV-uninfected (13/167 = 7.8%) men (p < 0.0001). Of 34 HIV-infected men with biochemical hypogonadism not on TRT, 11 (32.4%) had normal TT, but low FT. Of 12 HIV-uninfected men with biochemical hypogonadism not on TRT, none were in this category (p = 0.04) – all had low TT.

**Conclusions:**

The prevalence of biochemical hypogonadism in our sample of HIV-infected men was approximately 10%, with a substantial proportion of these men having a normal TT, but low FT. The measurement of AM FT, rather than TT, in the assessment of hypogonadism in HIV-infected men will likely increase diagnostic sensitivity and should be recommended.

## Background

Hypogonadism is common among HIV-infected men, even among men receiving antiretroviral therapy (ART), with prevalence estimates ranging from approximately 20% to 70% [1-3]. The cause of low testosterone (T) in HIV infection is multifactorial, and has been associated with poor clinical or nutritional status, use of certain prescription/illicit drugs (including opiates and methadone), pituitary dysfunction, HCV co-infection, advancing age, and changes in body composition [3-5]. Men with clinical symptoms of hypogonadism, including fatigue, weight loss, loss of libido or erectile dysfunction, depressive symptoms, or evidence of reduced bone mineral density, should undergo a laboratory evaluation for low T.

The Endocrine Society recommends morning (AM) measurement of total T (TT) as the initial diagnostic test and subsequent confirmatory test for hypogonadism [6]. Since elevated sex hormone-binding globulin (SHBG) levels result in higher TT levels, and SHBG is elevated in HIV-infected men [7,8], the guidelines further recommend free testosterone (FT) measurement for men in whom elevated SHBG is suspected [6], including men with HIV. In addition to having elevated SHBG, HIV-infected men may have inadequate T production, and as a result of these two factors, FT (the approximately 2% of T unbound or free to enter cells to exert metabolic effects) may be below the lower limit of normal in HIV-infected men even with a normal TT. Although FT is measured routinely for hypogonadism screening in some HIV clinics [9], the evidence for this practice has not been well documented. Limited clinical data has been available to support recommending FT measurement in HIV-infected men [10], and the current HIV primary care guidelines currently recommend measurement of TT for hypogonadism diagnosis [11]. Free T measurement in HIV-infected men has been recommended by endocrinologists for over ten years [5,12], however, there may be low uptake of this recommendation among HIV clinicians, particularly among those who were not practicing early in the HIV epidemic when hypogonadism in HIV disease was initially described [13].

The objectives of our study were to determine the prevalence of hypogonadism among a group of men who participate in the Multicenter AIDS Cohort Study (MACS) and to compare by HIV serostatus biochemical hypogonadism diagnoses made by TT and FT assays.

## Methods

This analysis used data collected in the MACS Cardiovascular Disease (CVD) substudy, which has been described previously [14]. Briefly, the substudy enrolled men older than 40 years who weighed less than 300 pounds and who had no history of coronary heart disease. Blood draws were performed at regularly scheduled MACS follow-up visits between April 2004 and January 2006. For this analysis, we included men who had undergone AM blood sampling for T measurement. Testosterone and SHBG were measured from archived sera using liquid chromatography-tandem mass spectrometry and radioimmunoassay, respectively, and assays were performed in Dr. Shalender Bhasin’s lab at Boston University. The testosterone assay had a sensitivity of 2 ng/dL, with an interassay co-efficient of variation ranging from 3.3% to 7.7% with three pooled samples analyzed in nine different assays. SHBG levels were measured using a two-site immunofluorometric assay (DELFIA-Wallac, Inc., Turku, Finland) [15,16]. The inter-assay CVs were 8.3%, 7.9%, and 10.9%, and intra-assay CVs 7.3%, 7.1% and 8.7%, respectively, in the low, medium, and high pools. The analytical sensitivity of the assays was 0.5 nmol/L. The assay has negligible crossreactivity with beta-microglobulin**,** thyroxine binding globulin (TBG), or corticosteroid binding globulin (CBG). Free testosterone was calculated using the Vermeulen equation [17].

The main outcome variable was biochemical hypogonadism, diagnosed by either FT < 50 pg/mL (< 173.5 pmol/L) or T < 300 ng/dL (< 10.4 nmol/L), consistent with guidelines [6]. Wilcoxon rank-sum (Mann–Whitney) and Fisher’s exact tests were used to compare demographic and clinical characteristics by HIV serostatus. Hormone values were log-transformed and adjusted by age, clinical site, BMI, and race. Pearson chi-square tests were used to compare by HIV serostatus both the prevalence of hypogonadism and the proportion of men with hypogonadism who had normal TT and low FT, whose biochemical hypogonadism would have been missed had only TT been measured.

## Results and discusion

### Results

**Figure 1:**
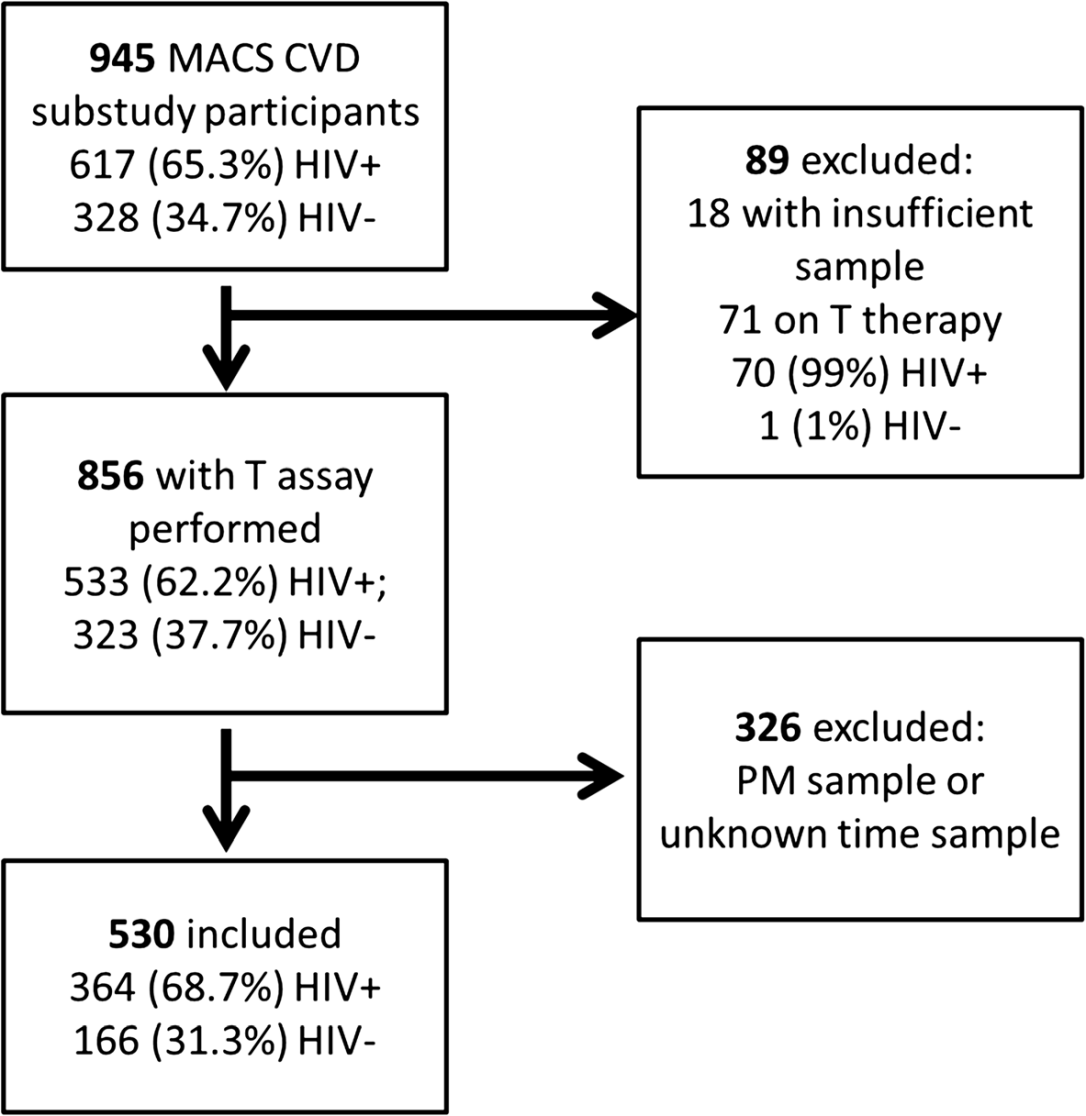
Study flow.

The study flow is shown in Figure 1. Of 945 men studied, T assays were not performed in 89 men, because of insufficient/no stored serum (n = 18) or because of receipt of T replacement therapy (TRT) (n = 71). Ninety-nine percent (70/71) of men excluded for receiving TRT were HIV-infected. There were 856 men who had a T assay performed, of whom 326 were excluded because their blood sample was drawn in the afternoon. Of the 530 men, 364 (68.7%) were HIV-infected and 166 (31.3%) were HIV-uninfected; Table 1 shows their demographic and clinical characteristics. The HIV-infected men were younger, weighed less, and were more likely to have hepatitis C virus (HCV) co-infection. Among the HIV-infected men, median nadir CD4 was 266 cells/mm^3^ and median current CD4 was 495 cells/mm^3^; 32% percent of the men were not virally suppressed (20.9% were not receiving ART at the time of the study).

**Table 1:** Demographic and clinical characteristics of study participants

	**HIV-uninfected (N = 166)**	**HIV-infected (N = 364)**	**p-value**
**Median Age (years)**	51.6	48.1	<0.001
**Race – White (%)**	68.7	64.3	0.32
**Median BMI (kg/m**^**2**^**)**	25.8	25.2	0.02
**Hepatitis C Virus Positive (%)**	7.8	14.6	0.03
**Median nadir CD4 cell count (cells/μL)**	---	266	---
**Median CD4 cell count (cells/μL)**	---	495	---
**HIV RNA ≥ 400 copies/mL**	---	32%	---
**Median Total T (ng/dL)**	650.4	629.2	0.43
**Median Free T (pg/mL)**	107.3	100.2	0.005
**Median SHBG (nmol/L)**	47.3	52.6	0.02

Median TT levels were not significantly different between HIV-infected and –uninfected men, while median FT was significantly lower and median SHBG was significantly higher in HIV-infected men (Table 1). When log-transformed hormone values were adjusted for age, BMI, race (white v. other), clinical site, and HIV serostatus, higher BMI (p < 0.0001) and white race (p = 0.01) were associated with lower log TT; older age (p < 0.0001) and HIV-infected serostatus (p = 0.001) were associated with lower log FT. Higher BMI and white race (p < 0.001 for both) were associated with lower log SHBG while advancing age (p = 0.01) and HIV-infected serostatus (p < 0.0001) were associated with higher log SHBG.

**Figure 2:**
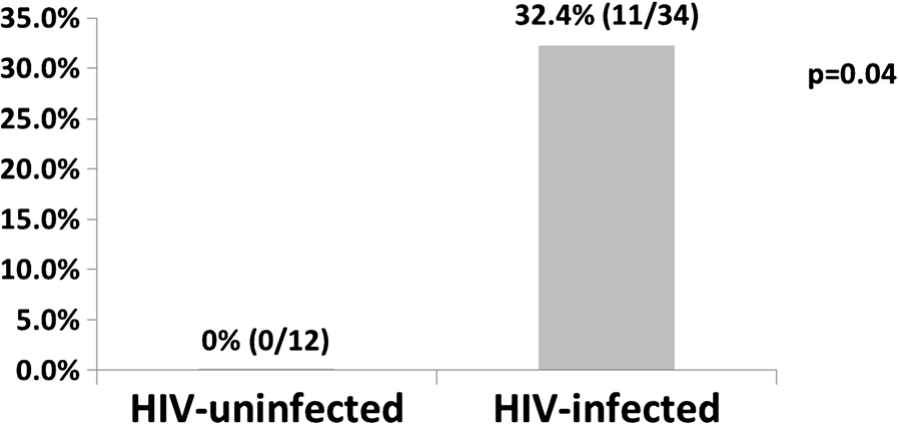
Proportion of hypogonadal men with normal T, low FT by HIV status.

The prevalence of biochemical hypogonadism, as defined by either low FT or low T, was similar in HIV-infected (34/364 = 9.3%) and HIV-uninfected (12/166 = 7.2%) participants (p = 0.42). Prevalence of hypogonadism, when men on TRT (n = 71) were included in the group of hypogonadal men, was higher in HIV-infected (104/434 = 24.0%) compared with HIV-uninfected (13/167 = 7.8%) participants (p < 0.0001). Of the 34 HIV-infected men with biochemical hypogonadism not on TRT, 11 (32.4%) had normal T, but low free T. In contrast, of the 12 HIV-uninfected men with biochemical hypogonadism, no men were in this category (p = 0.04) – all had low TT (Figure 2).

Among the 46 men with biochemical hypogondadism, we compared the proportion with normal TT and low FT with men who had low TT and either low or normal FT. Among the HIV-infected hypogonadal men, a full third of them had normal T, but low FT (Table 2). Therefore, if TT only had been used for diagnosis, about one-third of hypogonadal HIV-infected men would have been missed. In an exploratory analysis of the 34 HIV-infected hypogonadal men, we compared the characteristics of men who had a normal TT but low FT with men who had a low TT and low FT. More men with normal TT were HCV-infected (63.6% versus 11.4%, p = 0.001). Median [IQR] SHBG was higher among hypogonadal men with normal T and low FT than hypogonadal men with low T and low FT (109.0 [81, 143.9] versus 30.2 [20.2, 55.5] nmol/L, respectively, p < 0.0001). There were no differences by race, CD4 status, plasma HIV RNA level, or antiretroviral therapy status.

**Table 2:** Proportion of men with biochemical hypogonadism with normal TT, Low FT by HIV status

	**HIV-infected**	**HIV-uninfected**
Normal TT, Low FT	11/34 (32.3%)	0/12 (0%)
Low TT, Low FT	23/34 (67.6%)	12/12 (100%)

## Discussion

Among men participating in the MACS CVD substudy for whom an AM blood sample was available for T assay, the overall prevalence of biochemical hypogonadism was 8.7% (9.3% among HIV-infected men and 7.2% among HIV-uninfected men). Prevalence of hypogonadism, when men on TRT (n = 71) were included in the group of hypogonadal men, was significantly higher in HIV-infected compared with HIV-uninfected men (24.0% v. 7.8%). In the men not on TRT, the diagnosis of hypogonadism would have been missed among one-third of HIV infected men if TT only had been measured, while no HIV-uninfected men would have gone undiagnosed. Our results underscore the importance of using free T for hypogonadism diagnosis among HIV-infected men.

Hypogonadism prevalence among HIV-infected men has been reported as high as 70% [1-3]. In our cohort, when including men on TRT, the prevalence of hypogonadism in the HIV-infected men was 24.0%. Prevalence varies widely depending on the hypogonadism definition used and the demographic and clinical characteristics of the cohort, which likely accounts for the differences observed between our findings and other groups. Of note, hypogonadism among HIV-infected men has remained common despite successful ART [1]. Advancing age, higher body mass index (BMI) [18] and HCV co-infection [3] are associated with hypogonadism among HIV-infected men, and contribute to the persistence of hypogonadism despite successful ART.

We performed exploratory analyses among the HIV-infected hypogonadal men, comparing those who had a normal TT but low FT with men who had a low TT. Our objective was to evaluate whether we could provide guidance to clinicians as to which HIV-infected men would require FT testing; we found that men co-infected with HCV were more likely to have normal TT but low FT levels. Hence, HIV/HCV-coinfected men may represent a population at particular risk for misdiagnosis if TT alone is used to ascertain the presence of hypogonadism.

Consistent with findings from prior studies [2,7,8] we found that SHBG levels were higher among HIV-infected men. The exact pathophysiologic mechanisms accounting for this finding are unclear. Based on analyses of this same cohort, we have previously reported that SHBG increases with increasing age, black race, and HCV infection and decreases with increasing BMI [8]. Elevations in TT levels that occur as a result of elevations in SHBG decrease the diagnostic utility of the TT assay among HIV-infected men.

There are several limitations to this study. A diagnostic evaluation of hypogonadism is usually initiated because a patient reports symptoms such as low libido, fatigue, and low energy. Hypogonadism is a clinical diagnosis, and therefore the significance of biochemical hypogonadism diagnosed by laboratory assays is unclear. Commercially available testosterone assays vary in quality, and our assays, performed at a preeminent lab, may not have generated results that are representative of those readily available to clinicians using commercial assays. It is unclear how well FT calculated by Vermeulen equation reflects equilibrium dialysis, which is the gold standard. In our analysis, men with samples drawn after 12 noon were excluded, with an unknown effect on the outcome. Men receiving T therapy, most of whom were HIV-infected, were excluded. We presume that these men were receiving T therapy for diagnosed hypogonadism. By excluding these men, we may have introduced a selection bias because they may have been diagnosed using a low TT assay alone, possibly resulting in enrichment of numbers of included men with normal T and low FT. Even in this scenario, however, the number of HIV-infected men whose hypogonadism would have gone undetected by using TT measurement alone would still have been 11% (rather than the 32.3% we found) if all of the men who were receiving TRT had both low T and low free T levels. This represents a rate of hypogonadism still significantly higher than observed among the HIV-uninfected men. Finally, luteinizing hormone/follicle stimulating hormone (LH/FSH) levels were not performed; therefore, we were unable to determine if hypogonadism was primary or secondary.

The use of TT levels assayed from morning specimens to diagnose hypogonadism among HIV-infected men may result in about 30% missed cases. Morning free T levels are more sensitive diagnostically and should be measured in all HIV-infected men in whom hypogonadism is suspected.

## Conclusions

The prevalence of biochemical hypogonadism in our overall sample was approximately 10%, and prevalence of hypogonadism in HIV-infected men, when men on testosterone therapy were included in the estimate, was 24%. Use of morning TT levels to diagnose hypogonadism in HIV-infected men results in approximately 30% missed cases. Morning free T levels are more sensitive diagnostically and should be measured in HIV-infected men in whom hypogonadism is suspected.

## Competing interests

F.J.P. has received honoraria from Gilead Sciences, Tibotec Pharmaceuticals, and Bristol Myers Squibb. T.T.B. has received honoraria from Bristol Myers Squibb, Gilead Sciences, Tibotec Pharmaceuticals, ViiV, and serves as a consultant to EMD-Serono and Theratechnologies.

## Authors’ contributions

TTB conceived of the study; TTB and AKM helped design the study. AKM and TTB acquired, analysed and interpreted the data. AKM drafted the manuscript, ASD, FJP, LAK, MDW and TTB have revised it critically for important intellectual content. All authors have given final approval of the version to be published.
